# Effect of Vitamin D3 on Depressive Behaviors of Rats Exposed to Chronic Unpredictable Mild Stress

**DOI:** 10.3390/biomedicines11082112

**Published:** 2023-07-26

**Authors:** Fatimah R. Al-Ramadhan, Mahmoud M. A. Abulmeaty, Mohammed Alquraishi, Suhail Razak, Maha H. Alhussain

**Affiliations:** 1 Department of Food Science and Nutrition, College of Food and Agriculture Sciences, King Saud University, Riyadh 11451, Saudi Arabia; 441204446@student.ksu.edu.sa; 2 Department of Community Health Sciences, College of Applied Medical Sciences, King Saud University, Riyadh 11362, Saudi Arabia; malquraishii@ksu.edu.sa (M.A.); smarazi@ksu.edu.sa (S.R.)

**Keywords:** vitamin D, depression, behavioral tests, chronic unpredictable mild stress

## Abstract

**Simple Summary:**

Depression is a psychiatric disorder that represents a growing issue worldwide. This mental disorder is considered a mood disorder characterized by deep sadness and lethargy. A healthy diet is linked to enhanced mental health, whereas a bad diet is linked to an increased risk of depression and anxiety. Several studies have reported a positive association between vitamin D deficiency and depression. Therefore, we aimed to examine the effects of vitamin D3 (VD3) on a rat model of depression, which is induced by chronic unpredictable mild stress (CUMS). Behavioral tests were used to measure changes in depressive behaviors, as well as the levels of corticosterone and vitamin D in the blood. The groups that received doses of vitamin D produced better results than the group that did not receive any treatment in some behavioral tests. Vitamin D also had a protective role in preventing an increase of the corticosterone hormone, as well as an effective role in preventing a decrease in the level of vitamin D serum in the blood. The results of this research suggest that VD3 has a protective effect against anxiety and depression produced by CUMS in rats.

**Abstract:**

Depression is a psychiatric disorder that negatively affects how a person feels, thinks, and acts. Several studies have reported a positive association between vitamin D (VD) deficiency and depression. Therefore, we aimed to examine the effects of intraperitoneal injection of VD3, fluoxetine (antidepressant), and a combination of VD3 + fluoxetine on a rat model of chronic unpredictable mild stress (CUMS). A total of 40 male Wistar rats (224–296 g) were divided into five groups (*n* = 8 each) as follows: (1) the control group, (2) the CUMS group, (3) the CUMS group that received vitamin D (10 μg/kg), (4) the CUMS group that received fluoxetine (5 mg/kg), and (5) the CUMS group that received both vitamin D (10 μg/kg) and fluoxetine (5 mg/kg). The CUMS model was produced by exposing rats to frequent social and physical stressors for 21 days. In addition, blood samples were collected to determine corticosterone and serum VD levels. Also, behavioral tests were conducted, including the sucrose preference test (SPT), the forced swimming test (FST), the tail suspension test (TST), the open field test (OFT), and the elevated plus maze test (EPM). Our results show that VD3 had effects similar to fluoxetine on the depressive behavior of the rats when measured by three behavioral tests, namely SPT, FST, and OFT (*p* < 0.001). Additionally, VD3 had a protective effect against depression similar to that of fluoxetine. Corticosterone levels were lower in the CUMS group that received vitamin D and the CUMS group that received both vitamin D and fluoxetine than in the CUMS group (*p* < 0.000). In conclusion, VD3 has a protective effect against anxiety and depressive behaviors produced by CUMS in rats.

## 1. Introduction

Depression is a psychiatric disorder that represents a growing issue worldwide [1]. Stress is considered a contributing factor to depression. In response to stress, a reduction in neurogenesis in the adult hippocampus can occur, and this is important in the etiology of depression, including observations of decreased hippocampal levels in patients with depression [2].

At present, numerous new antidepressant medications have been introduced. However, response rates have not been enhanced, and the treatment of depression is still unsatisfactory. One of the leading reasons behind treatment failure in clinical practice is reduced patient compliance with antidepressant medications owing to their side effects [3]. Therefore, many nutritional and dietary components for the treatment of depression have been demonstrated. Indeed, precursors to neurotransmitters, including amino acids, omega-3 fatty acids, and some vitamins, are the most common nutritional deficiencies seen in mental disorder patients [3].

Vitamin D status is a vital determinant of general health and has been reported as a factor that might have positive health impacts on the treatment and prevention of various chronic diseases [4]. In addition, vitamin D’s significant importance in the brain’s processes, such as neuroplasticity and neuroimmunomodulation, suggests that it may have a role in psychiatric disorders, including depression. The biological plausibility of the relationship between depression and vitamin D has been supported by the recognition of vitamin D receptors in areas of the brain implicated in depression [5].

Several studies have reported a positive association between vitamin D deficiency and depression. A previous study conducted by Hoogendijk et al. [6] that examined older adults in the Netherlands reported that vitamin D concentrations were 14% lower in those diagnosed with depression. Another study examined the association between vitamin D concentrations at baseline and subsequent depression in a six-year prospective study of older adults and found that individuals with low vitamin D concentrations at baseline had significantly greater depression scores at the three- and six-year follow-up points [7]. Another study in a community of older men examined the retrospective and prospective associations between vitamin D concentration and depression and observed that a vitamin D concentration of less than 50 nmol/L was associated with higher odds of current, but not past, depression [8]. Furthermore, Rhee et al. [9] reported that serum 25-hydroxy vitamin D levels were inversely associated with cognitive/affective symptoms of depression only in Korean men. On the other hand, a study conducted among middle-aged and elderly Chinese community residents failed to find evidence linking depressive symptoms to vitamin D concentrations [10]. Taken together, the development of an effective delivery method for vitamin D could give promising results in managing depression. Thus, for the present study, we used a rat model to examine the effects of intraperitoneal (IP) VD3 doses on depression. We hypothesized that IP VD3 doses could improve behavioral markers of depression. This study aimed to examine the effects of IP doses of VD3, fluoxetine, and a combination of VD3 + fluoxetine on depressive behaviors and corticosterone levels.

## 2. Materials and Methods

### 2.1. Study Animals

A total of 40 adult male Wistar rats (224–296 g) aged 8–12 weeks were purchased from the animal house of the College of Pharmacy at King Saud University, Riyadh, KSA. The rats were housed in cages (45 × 25 × 15 cm), where they remained for one week for acclimatization. Standard animal care conditions were applied, including thermal zone temperature, humidity, and 12-h light/dark cycle. In addition, the rats had free access to regular rodent chow and ad libitum water. 

### 2.2. Ethical Considerations

The experimental protocol of the present study was approved by the Committee of Animal Research Ethics at King Saud University (No.: KSU-SE-22-24).

### 2.3. Experimental Design

The rats were divided into 5 groups, each consisting of 8 rats. The rats were randomly assigned as follows: (1) the control group, which was not exposed to any stress and was injected with 0.5 mL saline via the intraperitoneal (IP) route; (2) the CUMS group, which was exposed to the CUMS protocol and IP-injected with 0.5 mL saline; (3) the VD3 + CUMS group, which was exposed to the CUMS protocol and received vitamin D (10 μg/kg); (4) the fluoxetine + CUMS group, which was exposed to the CUMS protocol and received fluoxetine (5 mg/kg); and (5) the VD3 + fluoxetine + CUMS group, which was exposed to the CUMS protocol and received both vitamin D (10 μg/kg) and fluoxetine (5 mg/kg). All injections were given daily for 3 weeks.

### 2.4. Drugs and Chemicals

Vitamin D3 (cholecalciferol; Arnet Pharmaceutical Corp., Davie, FL, USA) was dissolved in 100% ethanol (1 μg/μL, stock) and diluted in 5% ethanol in distilled water for intraperitoneal (IP) injections [11,12] at a concentration of 10 μg/kg. Injections took place daily. Fluoxetine (Al-Qassim Pharmaceutical Plant, Saudi Pharmaceutical Industries & Medical Appliances, Buraydah, Saudi Arabia) was dissolved in physiological saline (0.9%) [13] and IP injections took place daily at a concentration of 5 mg/kg.

### 2.5. Chronic Unpredictable Mild Stress Model

The present study examined depression in experimental animals by following (with only minor modifications) the steps laid out by Ducottet et al. [14] for establishing a CUMS model. Herein, rats were exposed to variable, mild, and unpredictable stressors that changed randomly during the 3-week course of the study [15,16]. For example, rats might be exposed to “social defeat” stressors for 30 min, “tilting the cage at 45°” stressors for 24 h, “swimming” stressors for 5 min, and “food and water deprivation” for 24 h [17]. Table 1 presents the overall scheme of the present study’s CUMS protocol.

### 2.6. Behavioral Tests

#### 2.6.1. Sucrose Preference Test (SPT)

Before and after the CUMS procedures, all rats were assessed according to the sucrose preference test (SPT). The rats were subjected to food (i.e., food and water) deprivation for 24 h before they gained free access to a tube containing 10 mL of sucrose solution and another tube with a similar volume of pure water. One hour later, the volumes of sucrose solution and water consumed by the rats were recorded. The SPT values were calculated in percentages according to the following equation: % sucrose preference = sucrose consumption/(sucrose consumption + water consumption) × 100 [18]. Any decrease in the intake of sucrose solution was an indication of anhedonia.

#### 2.6.2. Forced Swimming Test (FST)

To test the modifications of depression-like behavior in the rats, we followed the forced swimming test (FST) and filled a clear cylinder (60 × 20 cm) to a depth of 30 cm with 25 °C water. On the first day, the rats were put individually in the cylinder and tested for 15 min. Then, the rats were dried and placed back in their cages until the next day. On the second day, the rats were examined for 5 min by a video camera. The two recorded parameters were (1) immobility time (floating in the water with only movements necessary to keep the head above water) and (2) swimming time (active swimming movements around the glass cylinder) [19]. Higher floating times were an indication of anhedonia.

#### 2.6.3. Tail Suspension Test (TST)

We performed the tail suspension test (TST) according to the guidelines laid out in previous publications with minor modifications [20]. Briefly, the rats were suspended by fixing a hook to their tails with adhesive tape. The hook was fixed about 1 cm from the tip of the tail and suspended 50 cm above the surface. The one recorded parameter was immobility, which was defined as no movement during the last 4 min of the 6 min test [20]. A decrease in movement was an indication of anhedonia.

#### 2.6.4. Open Field Test (OFT)

For the open field test (OFT), we placed the rats in the center of a square open field and observed them for 5 min using a video camera. Motor activity, rearing behavior, and grooming behavior were recorded for 300 s [21]. Lower times spent in the central region of the open field indicated anhedonia.

#### 2.6.5. Elevated Plus Maze Test (EPM)

The elevated plus maze test (EPM) assesses anxiety-like behavior in rats. The maze was made of grey Plexiglas and consisted of 4 arms (50 × 10 cm each), 2 with 40 cm dark walls and 2 with 0.5 cm ledges. For testing, rats were placed individually into the central portion of the maze facing a closed arm and removed after 5 min. The number of entrances and the time spent in the open or closed arms were recorded with the help of a video camera [21]. Lower times spent in the open arms indicated anhedonia.

### 2.7. Blood Collection and Analysis

After completion of the experiment, the rats were sacrificed, and samples of blood were withdrawn via cardiac puncture. Blood samples were centrifuged (New Brunswick, Ultra-Low Temperature Freezer; Eppendorf Company, Hamburg, Germany) at 3500 rpm for 10 min, and the serum was stored at −80 °C awaiting further analysis. The serum of 25-OH-VD was assessed using a commercial kit (Rat 25 Dihydroxy Vitamin D ELISA Kit, My BioSource, San Diego, CA, USA; catalog number MBS2601819). Corticosterone serum was also assessed using a commercial kit (Rat Corticosterone ELISA Kit, My BioSource, USA; catalog number MBS761865). All analyses were performed according to manufacturer protocols.

### 2.8. Statistical Analysis

Data were expressed as means ± standard deviation (SD). A value of *p* < 0.05 was considered statistically significant. SPSS version 23.0 (SPSS Inc., Chicago, IL, USA) was used for all statistical analyses. Using the Shapiro–Wilk test, we tested the variables for normality. As for between-group comparisons, we performed a one-way ANOVA with a post hoc test (Tukey test). For before-after comparisons, we performed the *T*-test or Wilcoxon signed-rank test according to the normality of the data. Correlation analysis of serum 25-OH-VD and behavioral tests was performed using Pearson’s correlation.

## 3. Results

### 3.1. Pre-Experiment Weight and Behavioral Tests

**Figure 1:**
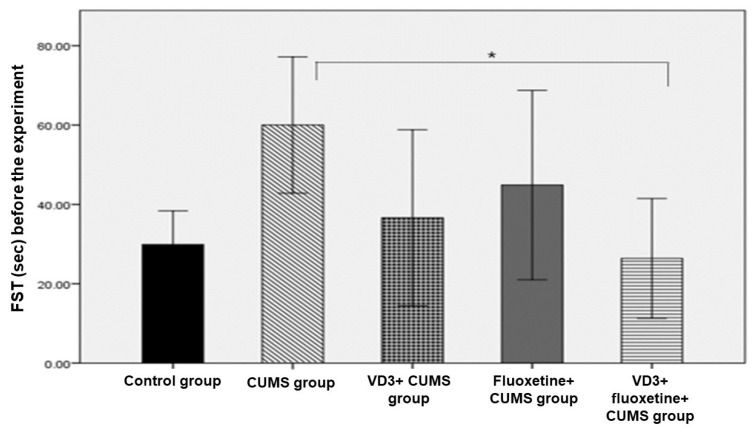
Forced swimming test across study groups before the experiment (*n* = 8). * *p* < 0.05 versus the CUMS group.

There were no significant differences in rat weight between the study groups. Also, the results of all pre-experiment behavioral tests yielded no significant differences between the groups, except for the FST. Regarding the FST, we noted a significant difference between the CUMS group and the VD3 + fluoxetine + CUMS group with a *p*-value of 0.030 (Figure 1).

### 3.2. Post-Experiment Weight and Behavioral Tests

**Figure 2:**
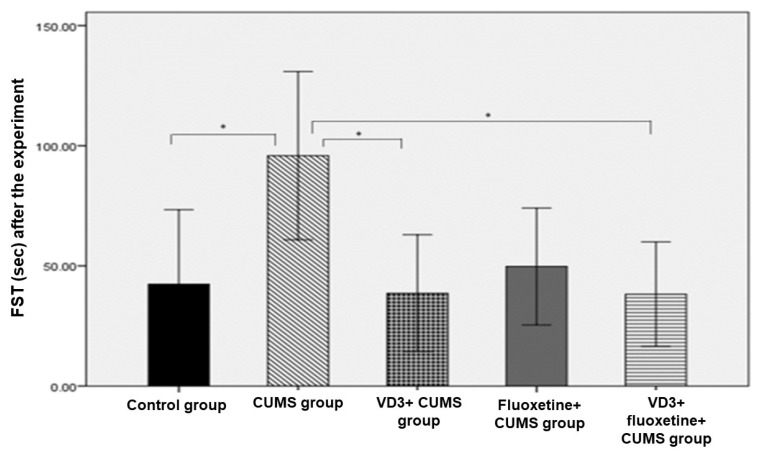
Post-experiment forced swimming test across study groups (*n* = 8). * *p* < 0.05 compared to the CUMS group.

**Figure 3:**
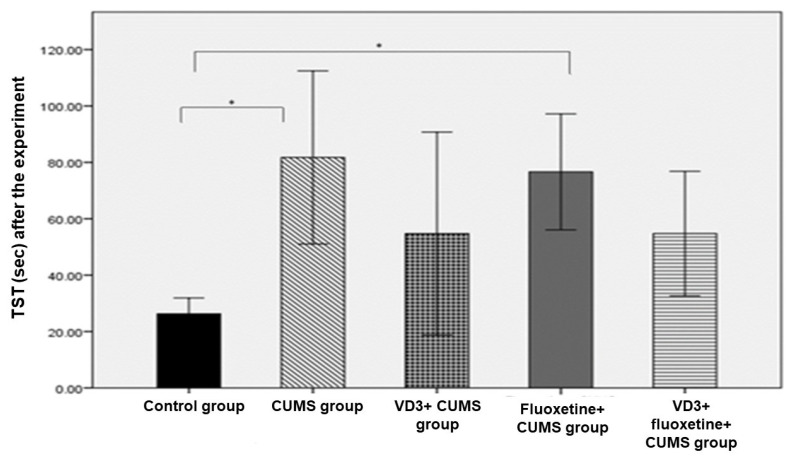
Tail suspension test across study groups after the experiment (*n* = 8). * *p* < 0.05 versus compared to the control group.

There were no significant weight-related differences among the five study groups. However, the results of the FST in the CUMS group were significantly different from the corresponding values for three other groups: the control group, the VD3+ CUMS group, and the VD3+ fluoxetine+ CUMS group. Also, the post-experiment TST results revealed a significant difference between the control group and the CUMS group, as well as between the control group and the CUMS + fluoxetine group (*p* < 0.007; Figure 2 and Figure 3).

### 3.3. Differences within Each Group

**Figure 4:**
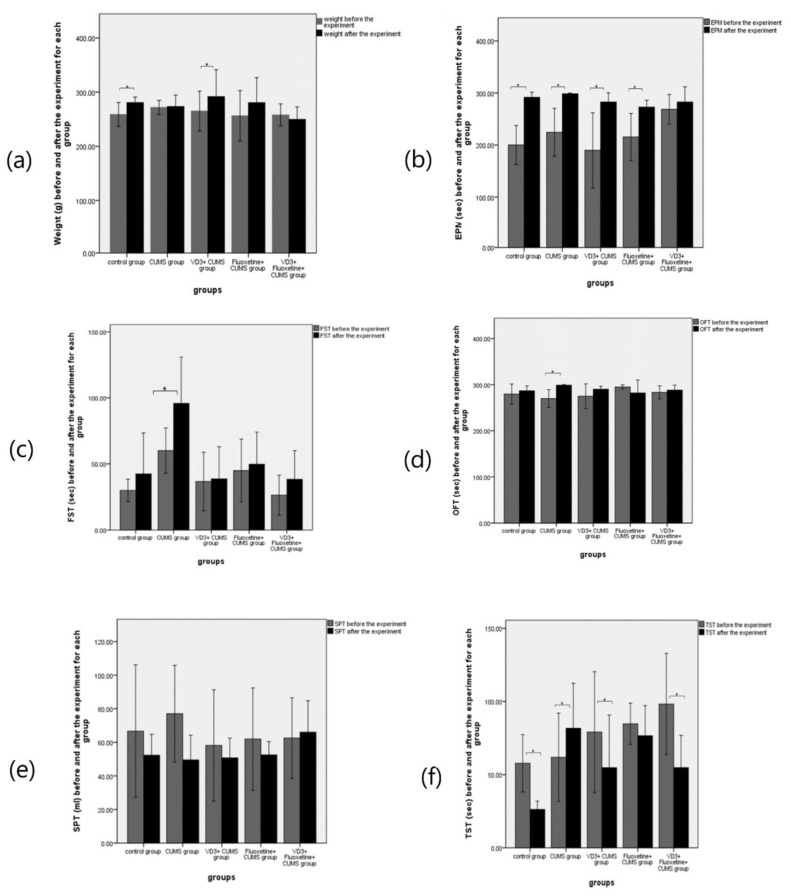
The effects of VD3, fluoxetine, and VD3 + fluoxetine injections on (**a**) weight, (**b**) elevated plus maze test (EPM), (**c**) forced swimming test (FST), (**d**) open field test (OFT), (**e**) sucrose preference test (SPT), and (**f**) tail suspension test (TST) (*n* = 8). * *p* < 0.05 versus the control group.

The results of the weight and behavioral tests before and after the experiment in each group are shown in Figure 4. We observed significant changes in weight, TST, and EPM (*p* < 0.05). However, the CUMS group underwent no significant change in weight. Most of the behavioral test results for the CUMS group revealed significant changes, including EMP, FST, OFT, and TST (*p* < 0.05). In the VD3 + CUMS group, significant weight changes were detected. Also, some tests for this group presented evidence of significant increases, such as EPM and TST (*p* < 0.05). Moreover, the fluoxetine + CUMS group did not have a weight change, with a *p*-value equal to 0.176, which is explained by the state of stress. For this group, only the EPM test presented evidence of significant change (*p* < 0.05). Regarding the VD3 + fluoxetine + CUMS group, the rats underwent no significant change in weight. As such, a combined treatment of VD3 and fluoxetine seems to prevent stress in most behavioral tests. 

### 3.4. Corticosterone Hormone and Vitamin D

**Figure 5:**
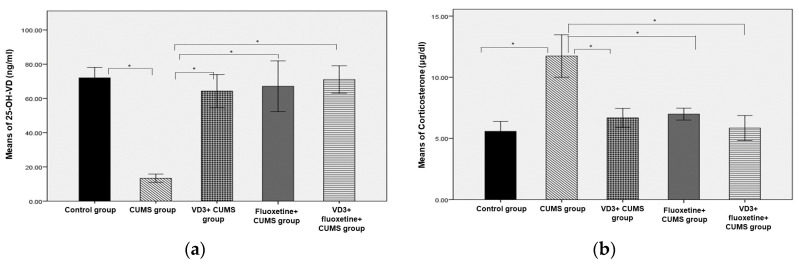
The effects of VD3, fluoxetine, and VD3 + fluoxetine on (**a**) serum vitamin D and (**b**) corticosterone hormone in a CUMS rat model. * *p* < 0.05 compared to the CUMS group.

Figure 5 shows the level of serum 25-OH-VD among study groups. The 25-OH-VD levels were significantly lower in the CUMS group compared to the other groups. Furthermore, the treated CUMS groups showed significantly higher 25-OH-VD, similar to the normal control group. Regarding levels of corticosterone hormone, the treated groups showed significantly lower levels than the CUMS group (Figure 5b).

### 3.5. Correlation Analysis of Serum 25-OH-VD and Behavioral Tests

Pearson’s correlation analysis for the relationship between serum 25-OH-VD and the behavioral tests showed a significant inverse correlation between the sucrose preference test and 25-OH-VD level in the VD3 + fluoxetine + CUMS group (Table 2). No further significant correlations were found between serum 25-OH-VD and the behavioral tests in the other studied groups.

## 4. Discussion

In this study, we examined the effects of IP doses of VD3, fluoxetine, and a combination of VD3 + fluoxetine on behavioral tests (sucrose preference test, forced swimming test, tail suspension test, open field test, elevated plus maze test) and serum corticosterone levels in a CUMS rat model. For three weeks, we followed CUMS procedures to generate stress and consequently depressive symptoms in the rats. Our results indicate that in both the VD3 + CUMS group and the VD3 + fluoxetine + CUMS group, the VD3 treatments had antidepressant effects, as evidenced by the results of three behavioral tests (FST, OFT, and SPT) and by a reduction in corticosterone hormone levels. Additionally, our findings show that, according to the results of the behavioral tests, the antidepressive effects attributable to the combination of VD3 and fluoxetine were better than the antidepressive effects attributable to individual treatment with VD3 or fluoxetine. Furthermore, in the VD3 + fluoxetine + CUMS group, an inverse correlation between the sucrose preference test and 25-OH-VD level was detected. Compared to the CUMS group, levels of the corticosterone hormone significantly decreased in all treated groups, indicating that amelioration of corticosterone is a crude mechanism for demonstrating the beneficial effects on depressive behaviors. Indeed, the increase in corticosterone in the CUMS group may have been caused by the reduction of serum VD.

In agreement with these findings, Bakhtiari-Dovvombaygi et al. [17] reported that treatment with VD 10,000 IU enhanced the performance of ovariectomized female rats in an OFT setting. These rats spent more time in the central area than rats from a CUMS group. Furthermore, FST results showed that both a CUMS groups treated with VD 1000 IU and 10,000 IU rats spent less time floating than CUMS rats without treatment. The current study is different in that it investigated only one dose of VD3 (400 IU) with or without fluoxetine rather than three doses (100, 1000, and 10,000 IU) and used male rats without any previous surgery. 

Our findings suggest that a combination of vitamin D and fluoxetine leads to better results than the use of one or other treatment alone, and this finding is in line with the evidence in Fedotova [22] that the combination of an antidepressant such as fluoxetine (10.0 mg/kg, IP) or paroxetine (10.0 mg/kg, IP) along with VD3 (5.0 mg/kg, subcutaneous injection) has impressive mitigating effects on depression-like behavior in long-term ovariectomized rats exposed to CUMS. The current study, however, used different doses of fluoxetine and VD, different injection routes, different duration, and different sex. Also, the previous study presented evidence suggesting that the coadministration of paroxetine and VD3 is more efficient than the coadministration of fluoxetine and VD3, even though the two antidepressants come from the same family of drugs, namely selective serotonin reuptake inhibitors (SSRIs). Paroxetine and VD3 had a greater mitigating effect on CUMS-induced behavioral, neurohormonal, and neuroimmunological impairments than paroxetine or VD3 by itself [22].

Several studies have shown that the procedures outlined in the CUMS model are generally associated with an increase in serum corticosterone levels [17,22,23]. In addition, other studies have established with a high degree of certainty that stress activates the hypothalamic-pituitary-adrenal (HPA) axis, increasing the levels of circulating glucocorticoids, such as corticosterone in rats and cortisol in humans [24,25,26]. This was observed in the present study; corticosterone levels increased in the CUMS group but not in any other groups (Figure 5b). Furthermore, we found that daily IP treatment with VD3 (10 μg/kg) prevented a rise in corticosterone levels as much as fluoxetine. Likewise, Sedaghat et al. [16] found that the combination of fluoxetine and VD (10 μg) significantly inhibited the elevation of corticosterone hormone levels. Recent reports suggested that depression can be considered a microglial disease [27], and Boontanrart et al. [28] found that vitamin D has a better mechanism for the prevention of depressive manifestations than SSRIs. They reported that VD3 induces changes in the activated microglia (during the disease condition), including reduction of proinflammatory cytokines IL-6, IL-12, and TNFα expression, as well as enhancement of IL-10 expression. Moreover, VD is incorporated into many pathways of neuroprotection, neurotransmission, and brain development [29].

Interestingly, it has been reported that a wide variety of vitamin D concentrations, employed under various experimental situations, can reduce depressive symptoms in a dose-dependent manner. A daily dose of 100, 1000, and 10,000 IU/kg of vitamin D was implemented with the highest dose being the most effective in reducing oxidative and inflammatory markers [17]. However, higher doses may generate adverse effects, especially in humans.

The association between VD and depression remains largely unexplored, but some plausible hypotheses have been presented. Indeed, the human brain has regions (e.g., the prefrontal cortex and hippocampus) that are known to be involved in the pathophysiology of depression and that contain not only vitamin D receptors but also the enzyme 1a-hydroxylase, which activates vitamin D, and so vitamin D might be involved in this process in some way [5,30]. Several other studies have pointed out that a cause of depression could be a lack of vitamin D [31,32,33]. Another possible explanation exists for the correlation between vitamin D deficiency and depression. In reverse, depression might lead to lower VD concentrations. This phenomenon is supported by the findings of Mulugeta et al. [34] and is consistent with our findings. In our study, the only group that exhibited a decline in vitamin D was the CUMS group; quite interestingly, there was no such decline in the CUMS + fluoxetine group, even though the group never received doses of vitamin D. Moreover, diet is unlikely to be a factor, as this was identical across all five groups and there was no significant difference between the pre- or post-experiment weights of all groups. Previous research has confirmed that taking supplements of different nutrients, such as vitamin D and omega-3 fatty acids, fails to stop or even slow the development of severe depressive episodes [35].

A limitation of this study is that it used only one dose of vitamin D treatment based on the frequently used less toxic dose in different biological conditions. A second limitation is the lack of molecular analysis, which could further our understanding of the effect of vitamin D in CUMS rats. Both genetic and signal transduction studies should be carried out to investigate further the link between vitamin D and depression.

## 5. Conclusions

In this study, we have presented a piece of evidence that vitamin D has an antidepressant effect, similar to that of pharmaceutical antidepressants (SSRIs). Vitamin D improved behavioral test scores, ameliorated corticosterone levels, and prevented potentially harmful decreases in serum vitamin D. Also, we found that a combination of vitamin D and SSRI antidepressants appears to have a desirable synergistic effect concerning depression and its symptoms.

## Figures and Tables

**Table 1:** Stressor plan based on the chronic unpredictable mild stress (CUMS) model.

Day	Week 1	Week 2	Week 3
One	Cage titling, 24 h	Swimming, 5 min	Cage titling, 24 h
Two	Tail pinching, 2 min	Restraint, 1 h	Tail pinching, 2 min
Three	Restraint, 1 h	Hot air, 10 min	Restraint, 1 h
Four	Hot air, 10 min	Tail pinching, 2 min	Hot air, 10 min
Five	Continuous light, 24 h	Continuous light, 24 h	Continuous light, 24 h
Six	Continuous light, 24 h	Continuous light, 24 h	Continuous light, 24 h
Seven	Water deprivation, 24 h	Food deprivation, 24 h	Food deprivation, 24 h

**Table 2:** Correlation of serum 25-OH-VD and the behavioral tests.

Variables	Control Group *n* = 8		CUMS Group *n* = 8		VD3 + CUMS Group *n* = 8		Fluoxetine + CUMS Group *n* = 8		VD3 + Fluoxetine + CUMS Group *n* = 8	
	r	*p*	r	*p*	r	*p*	r	*p*	r	*p*
EPM (s)	0.605	0.112	−0.311	0.453	0.011	0.979	−0.162	0.702	0.008	0.985
FST (s)	−0.039	0.927	0.136	0.748	0.079	0.852	−0.24	0.567	0.165	0.696
OFT (s)	0.524	0.182	−0.072	0.865	−0.252	0.547	−0.32	0.44	0.424	0.295
SPT (mL)	0.245	0.559	−0.566	0.144	0.047	0.912	−0.335	0.417	−0.707	0.049
TST (s)	−0.641	0.087	−0.467	0.243	−0.395	0.333	0.59	0.124	−0.387	0.344

*p*-value is tested by Pearson’s correlation. EPM, elevated plus maze test; FST, forced swimming test; OFT, open field test; SPT, sucrose preference test; TST, tail suspension test.

## Data Availability

Data supporting these results are available on reasonable request.

## References

[1] WHO (2008). Mental Health Gap Action Programme: Scaling Up Care for Mental, Neurological, and Substance Use Disorders. https://apps.who.int/iris/handle/10665/43809.

[2] Eisch A.J., Cameron H.A., Encinas J.M., Meltzer L.A., Guo L.M., Overstreet-Wadiche L.S. (2008). Adult neurogenesis, mental health, and mental illness: Hope or hype?. J. Neurosci..

[3] Lakhan S.E., Vieira K.F. (2008). Nutritional therapies for mental disorders. Nutr. J..

[4] Anglin R.E., Samaan Z., Walter S.D., McDonald S.D. (2013). Vitamin D deficiency and depression in adults: Systematic review and meta-analysis. Br. J. Psychiatry.

[5] Eyles D.W., Smith S., Kinobe R., Hewison M., McGrath J.J. (2005). Distribution of the vitamin D receptor and 1α-hydroxylase in the human brain. J. Chem. Neuroanat..

[6] Hoogendijk W.J., Lips P., Dik M.G., Deeg D.J.H., Beekman A.T.F., Penninx B.W.J.H. (2008). Depression is associated with decreased 25-hydroxyvitamin D and increased parathyroid hormone levels in older adults. Arch. Gen. Psychiatry.

[7] Milaneschi Y., Shardell M., Corsi A.M., Vazzana R., Bandinelli S., Guralnik J.M., Ferrucci L. (2010). Serum 25-hydroxyvitamin D and depressive symptoms in older women and men. J. Clin. Endocrinol. Metab..

[8] Almeida O.P., Hankey G.J., Yeap B.B., Golledge J., Flicker L. (2015). Vitamin D concentration and its association with past, current and future depression in older men: The Health In Men Study. Maturitas.

[9] Rhee S.J., Lee H., Ahn Y.M. (2020). Serum Vitamin D Concentrations Are Associated With Depressive Symptoms in Men: The Sixth Korea National Health and Nutrition Examination Survey 2014. Front. Psychiatry.

[10] Pan A., Lu L., Franco O.H., Yu Z., Li H., Lin X. (2009). Association between depressive symptoms and 25-hydroxyvitamin D in middle-aged and elderly Chinese. J. Affect. Disord..

[11] Trinko J.R., Land B.B., Solecki W.B., Wickham R.J., Tellez L.A., Maldonado-Aviles J., de Araujo I.E., Addy N.A., DiLeone R.J. (2016). Vitamin D3: A role in dopamine circuit regulation, diet-induced obesity, and drug consumption. Eneuro.

[12] Han H., Cui M., You X., Chen M., Piao X., Jin G. (2015). A role of 1, _25_(OH)_2_D_3_ supplementation in rats with nonalcoholic steatohepatitis induced by choline-deficient diet. Nutr. Metab. Cardiovasc. Dis..

[13] First M., Gil-Ad I., Taler M., Tarasenko I., Novak N., Weizman A. (2011). The effects of fluoxetine treatment in a chronic mild stress rat model on depression-related behavior, brain neurotrophins, and ERK expression. J. Mol. Neurosci..

[14] Ducottet C., Griebel G., Belzung C. (2003). Effects of the selective nonpeptide corticotropin-releasing factor receptor 1 antagonist antalarmin in the chronic mild stress model of depression in mice. Prog. Neuro-Psychopharmacol. Biol. Psychiatry.

[15] Burstein O., Franko M., Gale E., Handelsman A., Barak S., Motsan S., Shamir A., Toledano R., Simhon O., Hirshler Y. (2017). Escitalopram and NHT normalized stress-induced anhedonia and molecular neuroadaptations in a mouse model of depression. PLoS ONE.

[16] Sedaghat K., Naderian R., Pakdel R., Bandegi A.-R., Ghods Z. (2021). Regulatory effect of vitamin D on pro-inflammatory cytokines and anti-oxidative enzymes dysregulations due to chronic mild stress in the rat hippocampus and prefrontal cortical area. Mol. Biol. Rep..

[17] Bakhtiari-Dovvombaygi H., Izadi S., Moghaddam M.Z., Hashemzehi M., Hosseini M., Azhdari-Zarmehri H., Dinpanah H., Beheshti F. (2021). Beneficial effects of vitamin D on anxiety and depression-like behaviors induced by unpredictable chronic mild stress by suppression of brain oxidative stress and neuroinflammation in rats. Naunyn-Schmiedeberg’s Arch. Pharmacol..

[18] Liu M.-Y., Yin C.-Y., Zhu L.-J., Zhu X.-H., Xu C., Luo C.-X., Chen H., Zhu D.-Y., Zhou Q.-G. (2018). Sucrose preference test for measurement of stress-induced anhedonia in mice. Nat. Protoc..

[19] Estrada-Camarena E., Fernández-Guasti A., López-Rubalcava C. (2004). Interaction between estrogens and antidepressants in the forced swimming test in rats. Psychopharmacology.

[20] Duan C.-M., Zhang J.-R., Wan T.-F., Wang Y., Chen H.-S., Liu L. (2020). SRT2104 attenuates chronic unpredictable mild stress-induced depressive-like behaviors and imbalance between microglial M1 and M2 phenotypes in the mice. Behav. Brain Res..

[21] Fedotova J., Pivina S., Sushko A. (2017). Effects of chronic vitamin D3 hormone administration on anxiety-like behavior in adult female rats after long-term ovariectomy. Nutrients.

[22] Fedotova J. (2020). Different Effects of Fluoxetine and Paroxetine Combined with Vitamin D in Ovariectomized Rats Exposed to Unpredictable Stress. Open Biol. J..

[23] Koshkina A., Dudnichenko T., Baranenko D., Fedotova J., Drago F. (2019). Effects of vitamin D3 in long-term ovariectomized rats subjected to chronic unpredictable mild stress: BDNF, NT-3, and NT-4 implications. Nutrients.

[24] Pan Y., Zhang W.-Y., Xia X., Kong L.-D. (2006). Effects of icariin on hypothalamic-pituitary-adrenal axis action and cytokine levels in stressed Sprague-Dawley rats. Biol. Pharm. Bull..

[25] Johnson S.A., Fournier N.M., Kalynchuk L.E. (2006). Effect of different doses of corticosterone on depression-like behavior and HPA axis responses to a novel stressor. Behav. Brain Res..

[26] Watson S., Mackin P. (2006). HPA axis function in mood disorders. Psychiatry.

[27] Wang H., He Y., Sun Z., Ren S., Liu M., Wang G., Yang J. (2022). Microglia in depression: An overview of microglia in the pathogenesis and treatment of depression. J. Neuroinflamm..

[28] Boontanrart M., Hall S.D., Spanier J.A., Hayes C.E., Olson J.K. (2016). Vitamin D3 alters microglia immune activation by an IL-10 dependent SOCS3 mechanism. J. Neuroimmunol..

[29] Dell’Isola G.B., Tulli E., Sica R., Vinti V., Mencaroni E., Di Cara G., Striano P., Verrotti A. (2021). The Vitamin D Role in Preventing Primary Headache in Adult and Pediatric Population. J. Clin. Med..

[30] Eyles D.W., Burne T.H., McGrath J.J. (2013). Vitamin D, effects on brain development, adult brain function and the links between low levels of vitamin D and neuropsychiatric disease. Front. Neuroendocrinol..

[31] Berk M., Sanders K.M., Pasco J.A., Jacka F.N., Williams L.J., Hayles A.L., Dodd S. (2007). Vitamin D deficiency may play a role in depression. Med. Hypotheses.

[32] Schneider B., Weber B., Frensch A., Stein J., Fritze J. (2000). Vitamin D in schizophrenia, major depression and alcoholism. J. Neural Transm..

[33] Oude Voshaar R., Derks W.J., Comijs H.C., Schoevers R.A., de Borst M.H., Marijnissen R.M. (2014). Antidepressants differentially related to 1, 25-(OH)_2_ vitamin D3 and 25-(OH) vitamin D3 in late-life depression. Transl. Psychiatry.

[34] Mulugeta A., Lumsden A., Hyppönen E. (2020). Relationship between serum 25 (OH) D and depression: Causal evidence from a bi-directional Mendelian randomization study. Nutrients.

[35] Milaneschi Y., Peyrot W.J., Nivard M.G., Mbarek H., Boomsma D.I., Penninx B.W. (2019). A role for vitamin D and omega-3 fatty acids in major depression? An exploration using genomics. Transl. Psychiatry.

